# Neuro-Oncological Perspectives on Cancer Stem Cell Biology in Glioblastoma: Implications for Resection, Recurrence, Targeted Therapy, and Other CNS Tumors

**DOI:** 10.3390/cells15050413

**Published:** 2026-02-27

**Authors:** Karen Salmeron-Moreno, Karthik Papisetty, Chris Donghyun Kim, Thomas McCaffery, Rommi Kashlan, John Theodore, Jennifer Minseo Kim, Josephine Buclez, Hithardhi Duggireddy, Justin Maldonado, Hugo Guerrero-Cázares, Gustavo Pradilla, Tomas Garzon-Muvdi

**Affiliations:** 1 Department of Neurological Surgery, Emory University, Atlanta, GA 30329, USAchris.kim3@emory.edu (C.D.K.); jennifer.kim3@emory.edu (J.M.K.);; 2 Neurosurgery Department, Mayo Clinic, Jacksonville, FL 32224, USA

**Keywords:** cancer stem cells, stem-like cells, glioblastoma, tumor microenvironment, niche

## Abstract

**Highlights:**

**What are the main findings?**

- Interconnected niches support cancer stem cells (CSCs) by promoting phenotypic plasticity and multi-mechanism therapy resistance.
- CSCs are found in the infiltrative zone beyond contrast-enhancing tumor margins.

**What are the implications of the main findings?**

- Longitudinal therapeutic outcomes are increasingly dictated by the success or failure of total CSC eradication.
- Sustained therapeutic response will likely require multimodal approaches.

**Abstract:**

Cancer stem cells (CSCs) are increasingly recognized as central drivers of tumorigenesis, therapeutic resistance, and recurrence across diverse malignancies. This review synthesizes our current understanding of CSC biology across CNS tumors, with a focus on glioblastoma, where stem-like cells are sustained by specialized and overlapping tumor microenvironmental niches. Perivascular, hypoxic, invasive, immunosuppressive, and extracellular matrix-associated niches cooperatively enforce stemness, metabolic adaptability, immune evasion, and phenotypic plasticity, enabling CSC persistence despite maximal surgical resection and standard-of-care therapy. Notably, CSCs extend beyond radiographically defined tumor margins and populate peritumoral regions, providing a biological basis for near-universal recurrence. Advances in multiparametric imaging, stem cell-based ex vivo and in vivo models, and single-cell and spatial profiling have refined insight into CSC heterogeneity, niche dependence, and treatment resistance. Together, these findings reframe therapeutic strategies, highlighting the need for function-preserving maximal resection and multimodal therapies that target both CSC-intrinsic pathways and their supportive microenvironments.

## 1. Introduction

The recognition of stem-like tumor cell populations as key drivers of tumorigenesis, therapeutic resistance, and disease recurrence has fundamentally shifted the scientific approach to addressing cancer [1]. Despite substantial advances in targeted and cell-based therapies, there is a lack of sustained and meaningful clinical responses in patients with malignant brain tumors, emphasizing the need to identify and eradicate strategic cell populations accountable for tumor persistence [2]. This challenge is evident in aggressive central nervous system (CNS) tumors, where inter- and intra-tumoral heterogeneity prompts dynamic modeling of the tumor microenvironment (TME) and promotes adaptive resistance mechanisms to standard and targeted approaches [3].

Cancer stem cells (CSCs) are increasingly implicated as central mediators of tumor initiation, treatment resistance, and relapse [4]. The CSC hypothesis posits that many cancers are hierarchically organized and maintained by a small population of cells with stem-like properties, promoting tumor survival and metastasis through self-renewal and plasticity [5]. Ongoing research increasingly focuses on understanding genetic and molecular differences between CSCs and ordinary stem cells (SCs), with the aim of improving targeted therapies while preserving tissue homeostasis [6].

This review synthesizes current concepts in CSC biology with a focus on glioblastoma (GBM) and outlines the central role of neuro-oncology in enabling CSC-focused discovery and therapeutic translation.

## 2. Cancer Stem Cell Biology in CNS Tumors

Neural SCs are multipotent progenitor cells whose proliferation and lineage commitment are strictly regulated by microenvironmental cues. During development, neural SCs differentiate into neurons, astrocytes, and oligodendrocytes; in the adult brain, sustained neurogenesis is largely restricted to the subventricular zone (SVZ) and the subgranular zone of the dentate gyrus [7]. Whereas neural SCs sustain homeostasis, CSCs support tumor maintenance within a specialized TME ruled by dysregulated fate control [8], aberrant niche interactions [9], and robust stress-response adaptations that allow tumor persistence under defiant conditions [10]. Although neural SCs and CSCs share core properties, including self-renewal capacity, multipotency-like differentiation programs, and conserved molecular signaling pathways, CSCs lack normal cell-cycle restrictions, exhibit enhanced asymmetric division, and possess the ability to actively suppress antitumor immune responses, contributing to their aggressive nature [11,12].

Early conceptual foundations of CSCs can be traced to 19th-century observations proposing that tumors arise from undifferentiated cellular precursors [13,14]. Subsequent studies in embryonal tumors and teratocarcinomas reinforced this notion, establishing that poorly differentiated progenitors may generate diverse malignant lineages [13]. While classical CSC theory posited a rare, stable, self-renewing, unidirectional hierarchical compartment in which tumor-propagating capacity persists after cytotoxic stress by generating rapidly proliferating progeny with limited differentiation potential [8,15], modern refinements to this framework unveiled a plasticity model, characterized by dynamic, bidirectional interconversion between CSC and non-CSC states, shaped by intrinsic regulatory programs and microenvironmental cues [3,16,17,18,19]. Current evidence supports a mixed model in which progression and recurrence arise from both pre-existing quiescent stem-like cells and therapy- or niche-induced CSC states [8,19,20,21,22,23]. The relative contributions of these sources appear to be modulated by anatomical niches and treatment context; however, the precise proportions and governing rules of these dynamics remain an active area of investigation [8,23,24,25].

Within CNS tumors, CSC biology has been most extensively characterized in GBM, the most common and aggressive primary malignant brain tumor, which is defined by marked cellular and molecular heterogeneity and near-universal recurrence despite multimodal therapy [26]. Glioma stem cells (GSCs) have been implicated in tumor initiation, maintenance, and adaptive therapeutic resistance through mechanisms such as enhanced DNA damage responses, metabolic adaptation, and active remodeling of the TME [26,27,28].

### 2.1. Tumor Microenvironment

The existence of spatially and functionally specialized niches within the tumor where CSCs dwell has become a central framework for explaining why cytoreductive therapy alone rarely cures malignant CNS tumors [29]. Together, these niches sustain transient stem-like states, facilitate immune evasion, and provide resistance to cytotoxic injury through coordinated signaling [12,30,31,32,33]. Another important aspect is the migratory and invasive properties of these cells [34].

Currently, five niches have been described in the literature: perivascular [35,36], hypoxic [36,37,38], invasive [36,39], immunosuppressive [36,39,40], and extracellular matrix (ECM)-associated niches [22,41].

#### 2.1.1. Perivascular Niche

Within the perivascular niche, CSCs preserve stemness through direct cell–cell interactions and paracrine signaling with endothelial cells, pericytes, and pathological vascular remodeling [22]. In GBM, aberrant angiogenesis is largely carried by vascular endothelial growth factor (VEGF) secreted from CD133^+^ CSCs, with additional contributions from endothelial and myeloid cells [36]. Endothelial-derived NOTCH ligands actively strengthen CSC self-renewal, while CXCL12–CXCR4 signaling helps retain CSCs within the vascular niche and facilitates invasion [42,43]. IL-8 and αvβ8 integrin-mediated TGFβ1 activation are complementary routes by which tumors reinforce CSC maintenance, boost immunosuppression, and remodel the ECM [44,45,46,47,48,49,50]. CSCs utilize collateral energy sources allowing them to bypass angiogenic pathways which enables their resistance to VEGF-targeted anti-angiogenic treatments [51,52].

#### 2.1.2. Hypoxic Niche

The hypoxic niche induces stem-like transcriptional states and functional resilience [53]. In GBM, this niche is classically denoted by pseudopalisading necrosis, characterized by hypercellular regions surrounding necrotic foci [36,54]. Necrosis formation is attributed to tumor-driven microvascular thrombosis and vaso-occlusion [55]. Rather than reflecting slow tumor growth, oxygen deprivation acts as a potent regulatory signal through stabilization of hypoxia-inducible factors (HIFs), notably HIF-1α and HIF-2α [54,56]. These transcriptional states intertwine with STAT3, EGFR/PI3K/AKT, IGF1R, IL-8, and alarmin-RAGE signaling to support CSC tumorigenic capacity, cellular plasticity, migration, invasion, and chemoresistance [57,58,59]. HIF-1α enhances CSC self-renewal in part through stabilization of NOTCH intracellular domains [60], whereas HIF-2α preferentially activates stemness-associated transcriptional states, including NOTCH-related and calcineurin-dependent pathways [61]. Both factors can also promote dedifferentiation toward stem-like states through SOX2-dependent mechanisms, reinforcing the concept of stemness as an adaptive and reversible cellular program [62].

#### 2.1.3. Invasive Niche

The invasive niche emerges at the convergence of anatomical conduits with microenvironmental gradients of oxygen, pH, immune cells, and ECM conformation, shaping invasion patterns and selecting for specialized migratory tumor subpopulations [36,63,64]. In the adult brain, the SVZ denotes a highly vascularized neurogenic region whose normal pro-stem cell conditions also create a permissive hideout for invasive GSCs [65,66]. Perivascular and SVZ-associated CXCL12 gradients engaging CXCR4/CXCR7 direct GSC migration along vascular and white-matter tracts leading to tumor recurrence [66,67,68]. Invasion is also achieved through coordinated dynamics centered on the Na^+^–K^+^–Cl^−^ cotransporter NKCC1, by modulating focal adhesion dynamics and cell contractility [34].

At the tumor edge, GSCs are exposed to heightened metabolic and oxidative stress, where fluctuating oxygen and nutrient gradients promote selection and plasticity for stem-like states [69,70]. Integrins α6β1, αvβ3, αvβ5, αvβ6 mediate adhesion to laminin- and vitronectin-rich matrices, where FAK-Src dual kinase complexes activate downstream effectors to regulate motility, proliferation, and survival [71,72,73]. Across multiple models, WNT/β-catenin, TGFβ-SMAD, and NOTCH pathways interlock with HIF signaling to withstand epithelial–mesenchymal-like plasticity that enables invasion [74,75,76]. Clinically, this translates as diffuse GSC infiltration beyond MRI tumor boundaries, undermining complete surgical resection attempts and contributing to recurrence despite adjuvant therapy [77,78,79].

#### 2.1.4. Immunosuppressive Niche

The immunosuppressive niche rises from vascular dysfunction and tissue damage signals that, combined with hypoxia and necrosis, reshape local immunity by stimulating infiltration of regulatory T-cells (Tregs) and recruitment of bone-marrow-derived monocytes that differentiate into tumor-associated macrophages (TAMs), as well as granulocytes that mature into tumor-associated neutrophils (TANs) [80,81,82,83]. This immune context suppresses antigen presentation and attenuates T-cell effector activity through convergent signaling via VEGF, macrophage colony-stimulating factor 1 (CSF1), CXCL12, IL-1β, IL-6/STAT3, TGF-β, and immune checkpoint pathways such as PD-1/PD-L1 [36,37,84,85]. Functionally, this niche offers CSCs protection from chemoradiation by maintaining a relatively quiescent state and supporting subsequent cell-cycle re-entry, thereby facilitating tumor regrowth and relapse [86].

#### 2.1.5. Extracellular Matrix-Associated Niche

The pathological ECM niche crafted by the tumor provides a dynamic and directive scaffold that helps stabilize CSCs through integrated biochemical and biomechanical cues [87,88]. The glycoprotein tenascin-C is a central component that accumulates in invasive and perivascular regions, boosting angiogenesis and tumor cell proliferation [89,90]. Likewise, brain-derived chondroitin sulfate proteoglycans uphold infiltrative behavior and reinforce tumor-initiating capacity [91]. Furthermore, TAM secretion of TGF-β leads to ECM remodeling through metalloproteinases MMP2/9 and ADAMTS proteases, which amplifies EGFR activity at invasive fronts [90,92,93,94]. In parallel, integrin signaling and increased matrix stiffness act synergistically to activate the YAP/TAZ pathway, stabilizing CSCs and their migratory potential [95,96]. 

**Figure 1:**
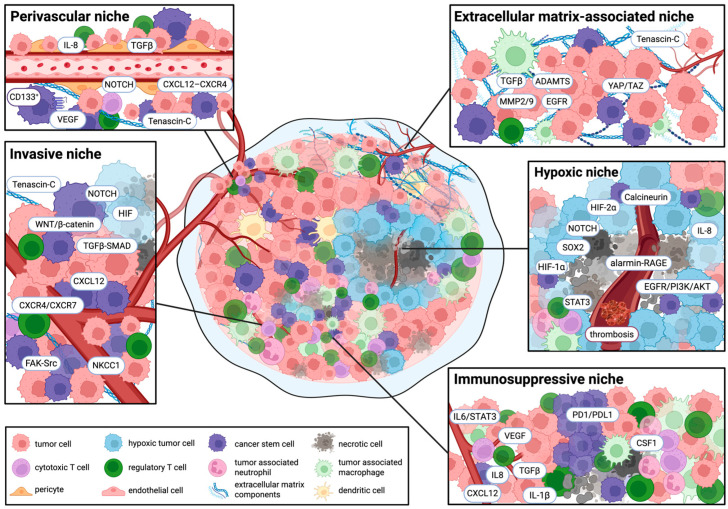
Integrated tumor microenvironment niches supporting cancer stem cell dynamics. Overview of the five tumor niches and the key molecular pathways through which they regulate cancer stem cell survival, self-renewal, and recurrence, Created in BioRender. Salmeron, K. (2026) https://BioRender.com/wyvf7h0.

These five categories overlap and should not be viewed as mutually exclusive [22]. Despite high vessel density, the chaotic nature of GBM vasculature creates intermittent perfusion that produces hypoxic gradients within the tumor [97], where immune cells and stromal elements integrate across microdomains, upregulating the CXCL12–CXCR4 signaling axis and thereby adapting to form an interactive migratory front and an immunomodulatory sanctuary for CSCs [22,43,67,98]. Niches are illustrated in Figure 1 and summarized in Table 1.

### 2.2. Other Brain Tumors

The CSC biology concept extends beyond GBM and appears broadly applicable to CNS tumors [27,28,99], with stem-like populations reported across adult and pediatric tumors, including meningiomas, pituitary adenomas, schwannomas, oligodendrogliomas, ependymomas, and medulloblastomas [99,100,101,102,103,104]. Although the depth of experimental validation varies among these tumors, accumulating evidence suggests that stem-like compartments may contribute to tumor initiation and recurrence [99].

Meningiomas are primary intracranial tumors that are typically benign [105]. The evidence base supporting distinct meningioma stem cells (MgSCs) remains less robust than in GSCs [106]. Nonetheless, developing data suggest the presence of stem-like populations expressing markers shared with GSCs, such as CD133, OCT-4, SOX2, and nestin [106,107,108]. Transcriptomic and signaling analyses further suggest that MgSC-associated signaling contributes to aggressive features and recurrence, with CXCL11 and CXCL12 implicated in malignant phenotypes [109]. Clinically, meningioma management is strongly influenced by tumor location: convexity tumors often permit wider surgical margins, whereas skull base tumors frequently abut or encase critical neurovascular structures, limiting the extent of resection [110,111]. Within these anatomical constraints, residual microscopic disease, and potentially MgSCs residing in protective niches, may persist following surgery and contribute to recurrence, particularly in higher-grade or surgically inaccessible tumors [101,106,112,113].

Pituitary neuroendocrine tumors (PitNETs) arise from anterior pituitary cells that are usually benign but can be locally invasive, recurrent, and treatment-refractory in a small subset of patients [114]. As in meningiomas, there is limited evidence for bona fide CSCs in PitNETs. Subpopulations expressing OCT-4, SOX2, CD133, and nestin have been identified in vitro and in xenografts, displaying proliferative capacity and multilineage differentiation toward hormone-producing pituitary cells, suggesting a potential role in local infiltration [102,115,116]. In an estradiol-benzoate-induced PitNET rat model, CSC markers were preferentially expressed in the adenoparenchyma rather than in the marginal zone, in contrast to their distribution in normal pituitary tissue [117]. Early evidence of PitNET CSCs suggests that these cells may migrate from marginal zones to form adenoparenchymal niches during early tumor development [117].

In medulloblastoma, the most common malignant pediatric brain tumor, CSC-like cells expressing CD133, SOX2, and nestin have been linked to tumor propagation, and resistance, with key developmental pathways including SHH, WNT, NOTCH, and MYC playing central roles in maintaining stemness [118,119,120]. A study using orthotopic medulloblastoma xenografts demonstrated that CD133^+^ tumor cells can form neurospheres and participate in multilineage differentiation even after several passages, suggesting the existence of CSCs in some medulloblastoma variants [121]. Ependymomas, particularly posterior fossa subtypes, also harbor stem-like, radial glia-like compartments shaped by profound epigenetic dysregulation, where TGF-β and WNT/β-catenin transcriptional states correlate with recurrence and poor clinical outcomes [122,123,124]. Single-cell analyses of all major ependymoma groups suggest that there are three fates of ependymoma stem-like cell differentiation: ependymal-like, glial-progenitor-like, and neuronal-precursor-like cells, with the most prognostically poor tumors mainly containing undifferentiated cells [122]. Across IDH-mutant oligodendrogliomas, progenitor-like oligodendrocyte precursor cell uses IDH-dependent methylation and chromatin changes to stall differentiation and enable plastic progression toward more proliferative progenitor states [125,126,127]. Though oligodendrocyte precursor cells have distinct origins from GSCs, a single-cell study of oligodendrogliomas revealed several overlapping functions contributing to proliferative capacity [127]. Schwannomas are benign neural crest-derived tumors that have been shown to express embryonic SC-like markers, including CD133, OCT-4, and SOX2, consistent with a stem-like compartment. The contribution of CSCs to schwannoma growth and recurrence is an important area for future functional work [128,129,130].

Despite broad evidence for stem-like features in many solid tumors, the extent of strict hierarchical organization and degree of CSC dependence differs across CNS tumors and even between molecular subtypes within a given histology [131,132,133]. Nonetheless, recurrent themes emerge, including activation of conserved developmental pathways, epigenetic plasticity, and niche-mediated protection from therapy [8,132,133].

Multiple potential biomarkers have been proposed for GSC identification, including CD133, CD44, CD15, SOX2, OCT-4, and nestin; however, these markers are neither universally expressed nor specific to GSCs, with substantial overlap across regular neural SCs and other tumor populations [134,135,136]. A persistent controversy is that marker-defined CSC fractions shift with culture conditions, microenvironmental cues, and therapy-induced stress, and marker positivity does not consistently align with tumor-initiating capacity; consequently, purely marker-based CSC estimates are difficult to interpret and challenging to compare across studies or tumor types [23,137]. Therefore, modern efforts increasingly integrate marker-based enrichment with functional assays and single-cell profiling [138]. Single-cell RNA sequencing has been leveraged to define transcriptional axes associated with classical and mesenchymal stem-like cellular states, including MEOX2-NOTCH and SRGN-NFκB programs [139]. With an abundance of regulatory signals, knowledge of key factors and behaviors of CSC molecular profiles is essential for understanding their highly heterogeneous nature [136]. Table 2 provides a comparative summary of markers across CNS tumor types.

## 3. Stem Cell-Based Research Models

Stem cell-based tumor models are needed to preserve the biological features that drive tumor persistence. These models retain characteristics of tumors including propagation, cellular plasticity, and clinically relevant heterogeneity [140]. By maintaining these dynamic and diverse states, they provide an essential framework to interrogate CSC biology and microenvironmental dependencies, enabling the identification of niche-derived cues that sustain CSC survival and tumor regeneration across ex vivo and in vivo approaches [141].

### 3.1. Ex Vivo Models

Ex vivo stem cell-based models enable controlled manipulation of niche-associated variables, allowing mechanistic understanding of CSC regulation [142]. Historically, neuro-oncology research has relied heavily on glioma-derived immortalized cell lines; although experimentally convenient, their growth as two-dimensional (2D) monolayers constrains cellular heterogeneity and microenvironmental gradients [143]. Furthermore, prolonged passaging often leads to clonal drift, yielding models that progressively diverge from the originating tumors [3,6,144]. Next-generation stem-like cultures incorporate ECM substrates, microfluidic gradient systems, and multicellular co-cultures to partially replicate structural and biochemical niche features and address the progressive divergence from progenitor cell lines [33,35,36].

Given the central role of three-dimensional (3D) architecture in glioma aggressiveness, 3D tumor models represent a major advance in neuro-oncology research [143,145,146]. Among these models, patient-derived tumor organoids (PDTOs) and neurosphere cultures established directly from surgical specimens and maintained under serum-free conditions can retain aspects of intratumoral heterogeneity linked to stemness and thereby mimic clinically relevant variability in therapeutic responses [6,11,144,147].

PDTOs preserve native cell–cell interactions and, in some cases, maintain infiltrating immune or stromal elements from the original tumor [11,86,143]. Organoids often exhibit selective radiosensitivity, with preferential elimination of differentiated tumor cells while adjacent GSCs persist, mirroring clinical patterns of incomplete eradication and relapse [143,148]. Additionally, the cerebral organoid glioma (GLICO) model provides a human neural-like scaffold in which GSCs infiltrate in patterns that closely resemble in vivo disease, forming tumor foci with microtube-associated networks implicated in infiltrative growth [149].

Despite these advances, organoids and neurospheres lack systemic physiology and therefore cannot fully portray aspects of tumor biology such as metastatic spread or drug pharmacokinetics [144]; in addition, cellular composition and architecture may vary depending on tumor sampling and culture conditions [3,6,150].

### 3.2. In Vivo Models

Whereas ex vivo models maximize experimental control, in vivo models uniquely capture tumor evolution, heterogeneity, and systemic interactions within the living brain [151,152]. Among CSC-relevant in vivo models, two principal methods emerge: orthotopic patient-derived xenografts (PDXs) and genetically engineered mouse models (GEMMs) [153].

PDXs, manufactured by implanting patient tumor cells or organoids into the brains of a typically immunodeficient host, give rise to tumors with strikingly patient-like features, including the histopathological hallmarks of high-grade glioma [143,144,154]. Consequently, PDXs display fundamental processes like diffuse infiltration into surrounding brain parenchyma and niche-like growth patterns associated with stem-like tumor populations, both of which are central to post-resection recurrence [154]. PDXs are a valuable translational bridge that allows for the assessment of drug delivery, systemic toxicity, and survival outcomes, attributes that are not readily accessible in vitro [155].

GEMMs of brain tumors are widely used to study CSCs and therapy responses in an immunocompetent setting [153,156]. Tumors can be initiated in situ within defined neural lineages using RCAS-tv-a somatic gene transfer, which restricts viral gene delivery to genetically specified cells; Cre-LoxP recombination, which enables conditional activation or inactivation of engineered alleles in selected cell types; transposon-based mutagenesis, which introduces insertional mutations to model multistep tumor evolution and facilitate driver discovery; or CRISPR/Cas9, which enables targeted somatic editing of candidate drivers [157]. Because these tumors arise within the intact brain and co-evolve with native immunity, GEMMs enable analysis of tumor-host interactions, treatment-induced immune responses, and post-surgical wound-healing processes, features unapproachable in immunodeficient systems [158].

Alongside ethical concerns, trade-offs for these models include long timelines, technical complexity, and imperfect alignment with human tumor genetics and heterogeneity. Nevertheless, in vivo models across multiple scales have been essential for examining growth patterns and surgical responses [159,160]. Ex vivo and in vivo models are summarized in Table 3.

## 4. Therapeutic Targeting of Cancer Stem Cells

Therapeutic targeting of CSCs in CNS tumors has become a major focus in translational neuro-oncology [161,162,163]. Therefore, current and emerging strategies aim to disrupt CSC intrinsic pathways, niche interactions and adaptive mechanism that underlie resistance to therapies [162].

### 4.1. Intrinsic Pathways

The strategy focuses on inhibiting conserved developmental signaling networks that sustain CSC self-renewal, survival, quiescence, therapy resistance and invasive potential [164]. Preclinical studies demonstrate that genetic or pharmacologic NOTCH inhibition through γ-secretase inhibitors reduces sphere formation, invasion, and self-renewal capacity in CD133^+^ glioma stem-like populations derived from U87 and U251 glioma cell lines, with downstream effects on AKT/mTOR [165,166,167]. Similarly, SHH pathway inhibition can reduce neurosphere formation and increase chemosensitivity [165,166,167]. WNT/β-catenin inhibitors have also been explored and shown to reduce stemness signatures and tumor-propagating capacity; however, clinical translation is complicated by the essential role of WNT signaling in normal neural progenitor regulation [165,166,167].

### 4.2. Chemokine Axis

Given the strong dependence of CSCs on microenvironmental cues, additional efforts aim to disrupt niche-mediated support [168]. Chemokine signaling through the CXCL12–CXCR4 axis exemplifies this strategy: CXCL12 produced by tumor vasculature and hypoxic regions recruits CXCR4-expressing GSCs, promoting survival and contributing to resistance to anti-angiogenic therapy such as bevacizumab [43,168]. In GBM models, CXCR4 upregulation correlates with CSC enrichment and tumor regrowth following VEGF blockade [169,170], whereas pharmacological inhibition of CXCR4 sensitizes tumors to radiotherapy and delays recurrence [171].

### 4.3. Immunotherapies

Harnessing immune responses to eliminate the CSC compartment has emerged recently as a strong approach [172]. Chimeric antigen receptor (CAR) T-cell therapy engineers autologous T-cells to recognize tumor-associated antigens enriched on CSCs [173,174]. In GBM, clinical efforts have focused on targets including IL-13Rα2, EGFRvIII, and HER2, among others [173,175]. Early-phase clinical studies of IL-13Rα2-directed CAR T-cell therapy have demonstrated proof-of-concept in recurrent GBM, although durable benefit remains limited by factors such as antigen heterogeneity and immune-excluded microenvironments; accordingly, current next-generation approaches focus on multi-antigen targeting, TME reprogramming, and improved CAR design [176,177,178].

### 4.4. Epigenetics

Multiple conserved epigenetic regulators implicated in stemness include chromatin and transcriptional co-regulators such as HDACs, BET/BRD4, EZH2/PRC2, BMI1/PRC1, and DNA methylation programs, all of which have promising pharmacologic inhibitors under investigation [179,180].

The variable and frequently limited therapeutic responses in GBM may be partly explained by therapy-induced selective pressures on CSCs and their niche interactions, which can drive unintended phenotypic shifts and redistribution into protected microenvironments [3,69,181]. For example, anti-angiogenic therapies targeting VEGF may disrupt perivascular support yet exacerbate regional hypoxia, promoting HIF-associated plasticity and a more aggressive, invasive phenotype [169,170]. In turn, radiotherapy, while effective against the proliferative bulk, may trigger CXCR4 upregulation and favor redistribution of surviving CSCs into relatively quiescent, therapy-shielded compartments [67,68,182]. Similarly, immunotherapies are frequently hindered by hypoxic and ECM-rich niches that foster immune exclusion by recruiting and potentiating myeloid-derived suppressor cells and reinforcing T-cell dysfunction [183,184].

Ultimately, CSC pathways are dynamic and reinforced specialized TME niches; therefore, therapeutic targeting of CSCs in CNS tumors is most likely to succeed as a multimodal strategy integrated with standard-of-care cytoreduction and chemoradiation [181,185]. Table 4 provides an overview of GBM CSC-directed approaches.

## 5. Surgical Implications of Cancer Stem Cells

The CSC paradigm defies the neurosurgical assumption that imaging-defined margins delineate the full biological extent of the disease [162,181,186,187]. Despite maximal resection of contrast-enhancing (CE) margins and adjuvant chemotherapy, GBM recurs within peritumoral MRI T2/FLAIR-hyperintense regions [188]. These areas harbor CSCs with the expression of stemness markers (SOX2, CD44 and CD133) at levels comparable to CE tumor, rendering macroscopic complete resection fundamentally insufficient and strongly associated with adverse outcomes [45,162,181,186,187,188,189,190,191,192]. Paradoxically, surgical intervention itself may create a favorable state for CSCs, as postoperative wound repair processes such as inflammation, angiogenesis, and reactive astrogliosis activate pleiotrophin-mediated pathways that promote CSC self-renewal and treatment resistance within the residual microenvironment [159,193].

Advanced multiparametric imaging reinforces this discrepancy by demonstrating hyperproliferative and metabolically active tumor extending well beyond CE margins, with non-enhancing volumes exceeding the primary tumor mass by up to 155%, with minimal spatial overlap observed on PET imaging [194,195]. Histopathologic correlation confirms comparable infiltrative tumor cell densities across both non-CE and CE regions [196]. Moreover, MRI-derived biomarkers, including low apparent diffusion coefficient and elevated relative cerebral blood volume, enable non-invasive localization of GSC-enriched regions [197].

Recurrence patterns in GBM are directly correlated with the extent of resection: local recurrence is the lowest following supramarginal resection, increases with gross total resection, and is the highest after subtotal resection [198]. However, this can be viewed as a double-edged sword, as distant relapse demonstrates the inverse trend, reflecting CSC-driven dissemination [198]. Recurrent tumors are not static replicas of the primary lesion but instead display adaptive cellular states at the infiltrative margin, where tumor cells exhibit neuronal or pluri-metabolic phenotypes, accumulate distinct mutational profiles, and show enrichment of immune-associated signatures [199], accompanied by upregulation of stem cell-related transcriptional programs including SOX2, OLIG2, POU3F2, and NOTCH1 [159].

**Figure 2:**
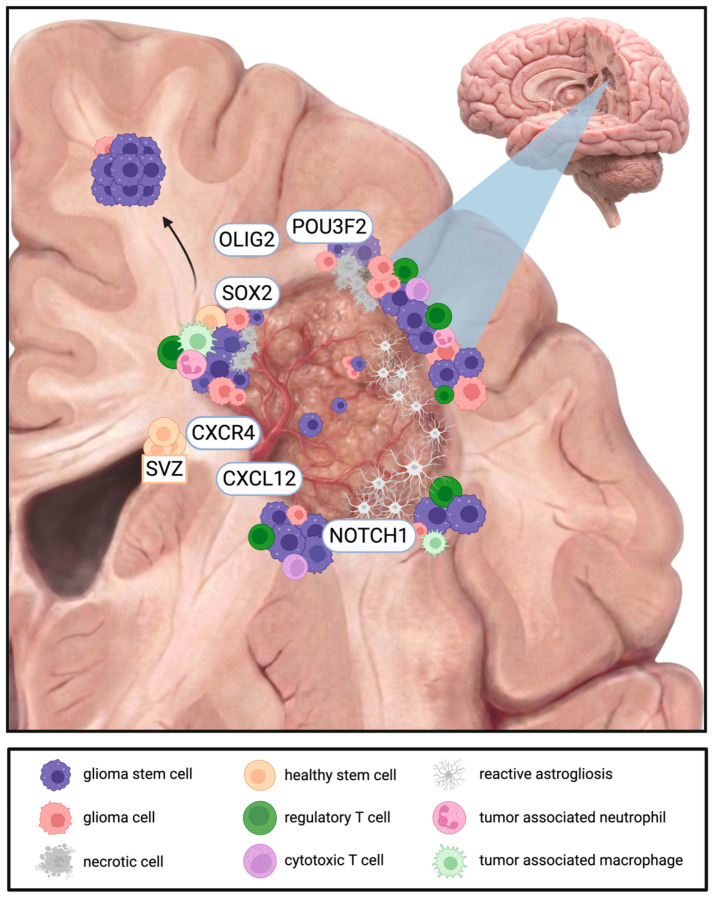
Cancer stem cell-driven mechanisms of local and distant recurrence in glioblastoma. Post-surgical resection cavity showing that residual stem-like populations can persist despite maximal resection and adjuvant therapy. CSC-like cells are shown across multiple spatial compartments, including the infiltrative tumor margin, perivascular and hypoxic niches, and along white-matter tracts. The subventricular zone (SVZ) is highlighted as a potential reservoir and conduit for tumor cell dispersal. Post-operative wound-healing signals promote local regrowth at the cavity edge and may also contribute to distal recurrence through seeding at remote sites, Created in BioRender. Salmeron, K. (2026) https://BioRender.com/u8iu2q4.

The niche contribution to these dynamics cannot be fully explained by a strict “local vs. distant” dichotomy. While local recurrence is broadly considered to be driven by perivascular and hypoxia-resilient CSCs within the resection cavity wall [200], these same populations appear to be conditioned by tumor–SVZ interactions [66,201]. The CXCL12–CXCR4 axis is considered to play a central role in this process by facilitating bidirectional trafficking, in which mutated cells migrate between the tumor and the SVZ, suggesting that the SVZ can reseed both the primary site and distant regions [43,66,68,201,202]. This is illustrated in Figure 2.

CSC insight has reframed surgical decision-making by balancing resection beyond traditional imaging margins while carefully preserving neurological function. More extensive resection confers a clear survival benefit in IDH-wildtype GBM compared to biopsy alone, and additional removal of non-CE tumor independently improves overall survival (HR 0.52–0.62, all *p* < 0.01) [203]. Current guidelines favor supramarginal resection incorporating T2/FLAIR abnormalities when safely feasible, particularly for patients with IDH-mutant GBM [204]. Meta-analyses further confirm significant improvements in overall and progression-free survival with both supramarginal and gross total resections when compared with subtotal resection [205,206]. Notably, survival benefit has been shown at 20% supramarginal extension, with more aggressive resection (>60%) providing no additional advantage [207].

Consequently, techniques enabling maximal safe resection are integral for advancing therapeutic outcomes. Awake craniotomy with cortical and subcortical mapping increases the extent of resection, reduces permanent deficits and improves progression-free survival outcomes [208,209]. Fluorescence-guided surgery with 5-ALA or fluorescein, combined with intraoperative MRI enhances visualization of infiltrative tumor beyond CE margins, and is associated with increased survival through extended cytoreduction [210]. These approaches acknowledge that, while complete CSC removal is currently unattainable, our evolving understanding of CSC continues advancing surgical outcomes.

## 6. Future Directions

As CSC biology becomes better defined, the field is gradually transitioning toward prospective, testable clinical-translational models that aim to map CSC-enriched microdomains and guide personalized therapies [211,212]. Shortened turnaround times for single-cell and spatial profiling now make it possible to return interpretable outputs within seven to fourteen days at specialized centers, fitting comfortably within the four-to-six week interval between GBM resection and initiation of adjuvant chemoradiation under the Stupp protocol, enabling correlation of compartment-specific CSC and niche features and paving the way for prospective clinical trial stratification [161,213,214].

A potential approach to examine CSC transcriptional states across GBM compartments is to integrate navigation-guided biopsies with intra-operative, region-matched sampling from the CE core, the T2/FLAIR infiltrative zone, and the peritumoral region, coupled with single-cell RNA sequencing, spatial transcriptomics, and histologic assessment [199,215]. Multi-region intra-operative sampling has been shown to be feasible in glioma workflows [216], and the RANO consortium’s consensus recommendations for imaging-defined tissue collection provide a practical framework for integrating research sampling with diagnostic pathology [217].

In addition to sampling, peri-operative window-of-opportunity trials for CSC-directed interventions involve selecting a candidate agent targeting an intrinsic pathway or niche interaction, administering it after diagnostic biopsy and before surgical resection, and analyzing the resected specimen for pharmacodynamics, shifts in CSC-marker expression, and changes in tumor architecture [218]. Proof-of-concept window trials for CSC have been established in other solid tumors, such as breast, colorectal, and non-small cell lung cancer, where short pre-operative exposure has demonstrated target engagement, supported biomarker development, and guided subsequent phase II trial design [219,220,221]. Applying this approach to GBM would provide an opportunity to evaluate CSC and niche dynamics under clinically realistic conditions while maintaining the standard of care.

Fulfilling this concept will depend on standardized, collaborative, and ethically grounded infrastructures, including clinically feasible timelines for functional modeling and equitable access to advanced profiling [222]. An appropriate workflow would need to include pre-operative coordination among neurosurgical, neuropathology, and research teams to define compartment targets and allocate specimens while preserving diagnostic priority [150], and immediate specimen preservation using standardized snap-freezing or other validated methods, with documentation of handling times and quality control [223,224].

## 7. Conclusions

CSC frameworks provide a biologically grounded explanation for persistence, adaptive resistance, and recurrence in GBM. Translational progress seems to be dependent on combining CSC-intrinsic targeting with niche-disrupting and immune-modulating approaches.

## Figures and Tables

**Table 1:** Summary of tumor microenvironment niches.

Niche	Key Signaling Drivers	CSC & Clinical Implications	References
Perivascular	VEGF, NOTCH, CXCL12-CXCR4, IL-8 and αvβ8 integrin-TGFβ1	Maintains CSCs stemness/self-renewal and survival; supports tumor propagation via angiogenic circuits/vascular remodeling	[36,43,44,45,46,47,48,49,50,51,52]
Hypoxic	HIF-1α, HIF-2α, STAT3, EGFR/PI3K/AKT, IGF1R, IL-8, alarmin-RAGE, NOTCH	Induces stem-like transcriptional states and functional resilience; promotes plasticity/dedifferentiation; increases migration/invasion and chemoresistance	[54,56,57,58,59,60,61,62]
Invasive	CXCL12-CXCR4/CXCR7 NKCC1 WNT/β-catenin, TGFβ-SMAD, NOTCH	Favors selection and plasticity for stem-like states; infiltration beyond radiographic margin undermines resection	[34,66,67,68,69,70,71,72,73,74,75,76]
Immunosuppressive	VEGF, CSF1, CXCL12, IL-1β, IL-6/STAT3, TGF-β, PD-1/PD-L1	Maintains CSCs in a quiescent state with later cell-cycle re-entry; chemoradiation resistance and relapse	[36,37,84,85]
ECM-associated	TGF-β, YAP/TAZ	Stabilizes CSCs; boosts angiogenesis and tumor cell proliferation; supports tumor infiltrating capacity and increased invasiveness	[90,92,93,94,95,96]

**Table 2:** Proposed cancer stem cell markers across CNS tumors, evidence base and clinical relevance.

Tumor	CSC Markers	Level of Evidence	Role	Clinical Implications	References
Glioblastoma	CD133, CD44, CD15, SOX2, OCT-4, Nestin,	Extensive in vitro, orthotopic xenograft, single-cell transcriptomics, strong clinical correlation	Functional tumor initiation and propagation, enhanced DNA damage response, metabolic adaptation, niche-dependent maintenance	Tumorigenicity, recurrence, invasiveness, migration, therapeutic resistance	[22,36,54,69,77,108,134,135,136]
Meningioma	CD133, OCT-4, SOX2, Nestin,	In vitro, transcriptomic/signaling analysis, clinical correlation	Stem-like marker expression shared with glioma stem cells, signaling pathways associated with aggressive phenotype and recurrence, anatomical constraints may permit persistence of stem cell niches after resection	Recurrence, aggressive features, persistence after subtotal resection	[106,107,108]
Pituitary Neuroendocrine Tumors	OCT-4, SOX2, CD133, Nestin	In vitro, xenograft, animal model (rat model)	Sphere formation, proliferative capacity, multilineage differentiation, estradiol-benzoate rat model showing CSC marker redistribution to adenoparenchyma	Local invasion, recurrence, treatment resistance	[102,115,116,117]
Medulloblastoma	CD133, SOX2, Nestin, CD33	In vitro, orthotopic xenograft, clinical correlation	CD133^+^ cells form neurospheres, retain multilineage differentiation across passages, developmental pathway activation linked to stemness and resistance	Tumor propagation, therapy resistance, recurrence	[118,119,120,121]
Ependymoma	Radial glia–like stem populations	Single-cell transcriptomics, clinical correlation	Epigenetically dysregulated radial glia–like compartments, three differentiation fates, undifferentiated states correlate with poor prognosis	Recurrence, poor clinical outcomes	[122,123,124]
IDH-mutant Oligodendroglioma	Progenitor-like oligodendrocyte precursor cell (OPC) states, IDH-dependent methylation/chromatin changes	Single-cell transcriptomics, genomic/epigenetic studies	IDH-driven methylation stalls differentiation, plastic shift toward proliferative progenitor states, overlap with GSC functional programs	Progressive proliferation, plasticity-driven progression	[125,126,127]
Schwannoma	CD133, OCT-4, SOX2	In vitro marker studies (functional contribution not yet established)	Embryonic Schwann cell–like marker expression, stem-like compartment suggested but limited functional validation	Potential role in growth and recurrence	[128,129,130]

**Table 3:** Comparative utility of ex vivo and in vivo CNS tumor models for cancer stem cell biology.

Model	Applications	Advantages	Limitations	Translational Readouts	References
Neurospheres	Stemness assays	Scalable, mechanistic clarity	Lacks microenvironment	Self-renewal capacity, drug IC50	[37,147,160]
Patient-derived organoids	Intratumoral heterogeneity, spatial CSC states, drug response, tumor–brain interactions	Preserves architecture	Partial microenvironment representation	Spatial transcriptomics, treatment response	[3,6,11,86,143,146,148]
Patient-derived xenografts	Drug delivery, pharmacokinetics, in vivo tumorigenicity	Preserves patient tumor genetics	Immunodeficient host, ethical concerns, does not fully mirror human genetics	Tumor growth delay, survival	[3,6,11,86,143,146,148]
Genetically engineered mouse models	Immune–CSC interactions, tumor initiation	Intact immune system	Heterogeneity, longer timelines, ethical concerns, does not fully mirror human genetics	Immune profiling, recurrence modeling	[153,156,157,158]

**Table 4:** GBM CSC-targeted therapies: targets, mechanisms and clinical status.

Target	Pathway	Aim	Development Stage	Examples	Clinical Trial ID
Angiogenesis	VEGF/VEGFR	Disrupt the perivascular niche, reducing access to oxygen and metabolic support	Phase 3	Bevacizumab for recurrent glioblastoma	NCT02511405
Invasion	CXCL12-CXCR4	Limit CSC trafficking to protective vascular niches, diminishing invasiveness	Phase 2	Plerixafor with modified radiation regimen	NCT03746080
Stemness	Notch/Hedgehog	Restrict self-renewal and maintenance of stem-like programs	Phase 2	Vismodegib for recurrent glioblastoma that can be removed by surgery	NCT00980343
Immune evasion	CAR-T	Direct targeting	Phase 1	IL13Rα2 CAR-T for recurrent or refractory glioblastoma	NCT02208362
	Vaccines		Phase 3	Rindopepimut for patients with newly diagnosed glioblastoma	NCT01480479
Hypoxic response	HIF-1α/HIF-2α	Disrupt the hypoxic niche that promotes radioresistance	Phase 1	Belzutifan for advanced solid tumors	NCT02974738

## Data Availability

No new data were created or analyzed in this study. Data sharing is not applicable to this article.

## Abbreviations

The following abbreviations are used in this manuscript:

CSCs	cancer stem cells
CNS	central nervous system
GLICO	cerebral organoid glioma
CAR	chimeric antigen receptor
CSF1	colony-stimulating factor 1
CE	contrast-enhancing
ECM	extracellular matrix
GEMMs	genetically engineered mouse models
GSCs	glioma stem cells
GBM	glioblastoma
MgSCs	meningioma stem cells
PDTOs	patient-derived tumor organoids
PDXs	patient-derived xenografts
PitNETs	pituitary neuroendocrine tumors
SCs	stem cells
SVZ	subventricular zone
3D	three-dimensional
TME	tumor microenvironment
TAMs	tumor-associated macrophages
TANs	tumor-associated neutrophils
2D	two-dimensional
VEGF	vascular endothelial growth factor
